# The Role of Quantitative EEG in the Diagnosis of Neuropsychiatric Disorders

**DOI:** 10.25122/jml-2019-0085

**Published:** 2020

**Authors:** Livia Livint Popa, Hanna Dragos, Cristina Pantelemon, Olivia Verisezan Rosu, Stefan Strilciuc

**Affiliations:** 1. “RoNeuro” Institute for Neurological Research and Diagnostic, Cluj-Napoca, Romania; 2. Department of Clinical Neurosciences, “Iuliu Hatieganu “University of Medicine and Pharmacy, Cluj-Napoca, Romania

**Keywords:** EEG, QEEG, Quantitative, Review

## Abstract

Quantitative electroencephalography (QEEG) is a modern type of electroencephalography (EEG) analysis that involves recording digital EEG signals which are processed, transformed, and analyzed using complex mathematical algorithms. QEEG has brought new techniques of EEG signals feature extraction: analysis of specific frequency band and signal complexity, analysis of connectivity, and network analysis. The clinical application of QEEG is extensive, including neuropsychiatric disorders, epilepsy, stroke, dementia, traumatic brain injury, mental health disorders, and many others. In this review, we talk through existing evidence on the practical applications of this clinical tool. We conclude that to date, the role of QEEG is not necessarily to pinpoint an immediate diagnosis but to provide additional insight in conjunction with other diagnostic evaluations in order to objective information necessary for obtaining a precise diagnosis, correct disease severity assessment, and specific treatment response evaluation.

## Introduction

Since 1929, when Hans Berger recorded the first electroencephalogram (EEG), the field of brain electrophysiology has seen significant progress. Berger's observations were limited to the time domain, but he suggested that frequency analysis would improve the interpretation of EEG signals in the future [1]. The utilization of computers for EEG analysis began in the 1970s, and Marc Nuwer defined digital EEG for the first time in 1997 [2]. Digital EEG provides multiple advantages, such as an easy selection of significant features for the correct acquisition of EEG, the possibility of modifying the sensitivity of parameters, and the frequency range in order to analyze only certain parts of the EEG signal, more precise and specific interpretation [3].

Furthermore, in the same report, Marc Nuwer introduced the concept of quantitative EEG (QEEG) [2]. QEEG stands for modern EEG analysis and involves the recording of digital EEG signals that are processed, transformed, and analyzed using complex mathematical algorithms. QEEG brought new techniques of EEG signals feature extraction: analysis of specific frequency band and signal complexity [4], analysis of connectivity, and network analysis [5]. In this article, we review the existing literature on the clinical applications of QEEG.

### Clinical applications of QEEG

#### Neuropsychiatric Disorders

The American Academy of Neurology (AAN) and the American Clinical Neurophysiology Society (ACNS) state that QEEG may be complementary to conventional EEG in the following situations: screening of possible epileptic peaks or seizures, screening of epileptic seizures in patients at risk that are admitted to an Intensive Care Unit (ICU), pre-surgical assessment in drug-resistant epilepsy, detection of acute intraoperative intracranial complications, evaluation of patients with cerebrovascular disease symptomatology, severity assessment of dementia and encephalopathies and ambulatory EEG [3]. On the other hand, in experimental studies with no evidence in clinical practice, QEEG is used for the following conditions: post-concussion syndrome, mild or moderate traumatic brain injury, attention deficit disorder, schizophrenia, depression, alcoholism, tinnitus and for monitoring the therapeutic response to psychotropic drugs [3].

#### Epilepsy

The EEG is a standard assessment tool in epilepsy. Although QEEG does not have the same widespread use as EEG, it can provide a rapid diagnosis of epileptic seizures and also differential diagnosis between different subtypes. Goenka et al. suggested that different types of seizures have specific QEEG patterns, increasing the sensitivity of their identification, and improving the diagnosis [6]. Thus, in their study, the sensitivity of QEEG spectrograms in seizure diagnosis was between 43% and 72%, and the asymmetry was correlated with focal seizures in 117 out of 125 patients with a sensitivity of 94% [6]. Another role of QEEG in epilepsy is to evaluate the response to antiepileptic therapy using pharmaco-EEG studies. According to the International Society of Pharmaco-EEG (IPEG), quantitative pharmaco-EEG is the description and quantitative analysis of the effects of substances on the central nervous system in clinical and experimental pharmacology, neuro-toxicology, therapeutic research and other disciplines [7]. Multiple studies on neuropsychiatric treatments have suggested the effects of drugs on the brain wave features so that EEG analysis becomes an essential tool in the classification of psychopharmacological agents [8]. Rosadini and Sannita [9] claim to be the first to apply QEEG in order to analyze the effects of anticonvulsants by studying spectral power in repeated EEG records for 16 months associated with plasma dosages of ethosuximide, diphenylhydantoin, valproic acid, and phenobarbital [8]. The most common identified effects were: EEG slowing, increase in delta (δ), and theta (θ) activity and decrease in the high-frequency bands, a slowdown in the dominant rhythm being specific [8]. Considering that cognitive impairment (CI) may occur in 70-80% of patients with epilepsy, CI evaluation through QEEG parameters could contribute to a better understanding of the pathophysiology of altered cognitive activity in epilepsy. A correlation between absolute power, inter- and intra-hemispheric coherence and cognitive activity in patients with epilepsy over 18 years has been suggested in some studies [10]. Absolute power was increased in all frequency bands in epileptic patients, and intra- and intra-hemispheric coherence in the θ band was higher in patients with epilepsy than healthy patients [10].

#### Stroke

Stroke patients usually present with typical cerebral rhythms abnormalities. QEEG in diagnosing or monitoring stroke abnormalities was first used in 1984, and the most remarkable result was that the θ/β ratio significantly increased in the damaged hemisphere [11]. Also, it was found that the healthy controls showed a very high degree of symmetry in all parameters [11]. α relative power was reduced both in the damaged and normal hemisphere [12], and post-stroke recovery may be evaluated using this pattern. Frontal α activity is associated with the functional outcome and progression of cognitive impairment because it may be an index of attentional capacity post-stroke [13]. The δ/α ratio (DAR) and α asymmetry index were also increased [12]. Recent studies suggested the utility of a ‘lower-density’ EEG electrode montage – just four frontal electrodes: F3, F7, F4, and F8 for assessing the diagnosis and monitoring in stroke [13].

Furthermore, the DAR measured in four frontal electrodes montage correlates with the neurological outcome in patients with anterior circulation stroke [14]. The Brain Symmetry Index (BSI) was initially used in monitoring potential cerebral ischemia during carotid surgery, but, in 2004, Van Putten and Tavy suggested that BSI could be a measure for the amount of ischemic damage [15]. BSI is ‘the mean of the absolute value of the difference in mean hemispheric power in a frequency range from 1 to 25 Hz’ according to their study [15]. Furthermore, a positive correlation between the NIHSS score and the BSI was reported [15].

#### Traumatic Brain Injury (TBI)

Advanced neuroimaging techniques have contributed to a better understanding of neuropathological mechanisms in TBI. Neuroimaging through Diffusion Tensor Imaging (DTI) has highlighted changes in functional connectivity between brain regions – evidence of white matter integrity damage in TBI [16]. Instead, by using Magnetic Resonance Spectroscopy (MRS), abnormalities of the cerebral metabolism have been shown as a consequence of TBI [16]. These molecular changes, visible on DTI and MRS, affect the generation, transmission, and processing of neural signals within and between brain regions [16]. Furthermore, studies have suggested a high correlation between DTI/MRS changes and abnormalities in cerebral electrical activity, suggesting the utility of EEG in assessing functional cerebral impairment [16].

It is important to emphasize that there are no specific EEG or QEEG patterns in TBI. The classification of EEG/QEEG changes in mild traumatic brain injury (mTBI) is presented in table 1. The majority of acute EEG changes disappear in about three months, and 90% during the first year after the trauma [17, 18]. The most common QEEG abnormalities reported in patients with TBI are: reduction of the mean α frequency [19–23], an increase of θ activity [24–27] and increase in θ/α ratio [20, 28, 29]. Other studies suggested changes in frontal and frontotemporal coherence and phase [30] and the severity index [31].

**Table 1:** Classification of EEG/QEEG changes in mTBI.

**Acute EEG/QEEG changes in mTBI**	Epileptic activity, followed by a 2-minute diffuse attenuation of cortical activity that returned to normal within 10 minutes to one hour [32, 33]
	Reduction of the mean α frequency [21]
	Increase in θ [24, 25]
	Increase in δ [19]
Increase of θ/α ratio [18, 23, 28]
Subacute EEG/QEEG changes in mTBI (weeks or months after mTBI)	Increase of 1-2 Hz of the posterior α rhythm was detected, explained by the normalization of EEG after the post-traumatic slowdown [17, 34]
Chronic EEG/QEEG changes in mTBI	Epileptiform changes at 16% of patients with psychiatric, cognitive or somatic symptoms developed in the first few weeks after mTBI [35]
	Slow-wave changes in 63% of the same patients [35]
	Increase in δ power in patients with post-concussion syndrome [18]
	Reduction in δ power in patients with post-concussion syndrome [18]

QEEG coherence and phase may detect and quantify the severity of mTBI and diffuse axonal injury [30]. The importance of these markers in diagnosing TBI has recently been demonstrated in studies showing that phase and coherence reflect topographical inhomogeneity associated with changes in cortical architectonic and axonal fibers [30, 36–38]. In addition to these observations, the results of a prospective study of 162 patients with severe, moderate, or minor TBI highlighted that phase and coherence were the best predictors of prognosis at one year after TBI [30, 39]. Thatcher used spectral power, coherence, and phase in order to assess the effects of mTBI, identifying the following changes in patients with a history of TBI: increase in frontal and frontotemporal coherence and decreased phase, reduction of the anterior-posterior spectral power differences and α power reduction in posterior regions [30].

In a recent study, the same researchers suggest that QEEG changes due to TBI may develop early and may remain detectable for a long time [31]. These changes can be evaluated through the TBI severity index with 96% accuracy, 95% sensitivity, and 97% specificity [31]. TBI severity index may predict the Glasgow score, the duration of post-traumatic coma, and the post-TBI performance in neuropsychological tests [31] retrospectively. However, this index has limited applicability in current clinical practice – studies of TBI patients with well-defined inclusion criteria are required, which may also take into account other neuropsychiatric comorbidities, drug administration, and other potential risk factors [31].

A study published in 2018 described the development and validation of a new index calculated with QEEG methods – Brain Function Index (BFI) [16]. Patients aged 18-85 years presented at the emergency room within 72 hours of a concussion with a Glasgow score of 12-15, were enrolled in the study. BFI turns out to be a quantitative marker of brain function impairment in TBI that may suggest the severity of the lesion and the prognosis of the patient with TBI [16]. In clinical practice, BFI could contribute to early diagnosis in TBI [16] and, thus, influence the onset of sequelae and subsequent complications. The BFI may identify functional brain damage in TBI that cannot be diagnosed with CT [16]. Thus, it provides objective information on the susceptibility of a functional cerebral deficit.

The data supported by the studies conducted so far on QEEG's contribution to the TBI offer the premise of the development of QEEG methods for TBI diagnosis. Further research will have a significant impact on increasing the confidence interval for the sensitivity and specificity of QEEG in the diagnosis and dynamic monitoring of TBI.

#### Encephalopathy

QEEG may highlight some neurophysiological aspects associated with an altered state of consciousness. Relative power in the α frequency band assesses the QEEG diagnosis of encephalopathy of different causes (Creutzfeldt-Jacob disease, uremia, hypoxic-ischemic encephalopathy) and also the differential diagnosis of delirium [40]. The most common parameters are θ activity, the relative power in δ frequency bands, and the activity in slow bands frequency [41–43]. According to the American Academy of Neurology recommendations of Classes II and III, QEEG analysis can be a handy tool, additional to conventional EEG, in cases of uncertain diagnosis of encephalopathy [2, 44].

#### Intensive Care Units

QEEG may be complementary to conventional EEG when an accurate diagnosis of the most discrete EEG abnormalities is needed. Studies on the utility of QEEG in Intensive Care Units (ICU) have analyzed the following pathological conditions: carotid endarterectomy, cerebrovascular interventions (for acute intracranial complications), situations in which cerebral blood flow is compromised in comatose patients [40]. The American Association of Neurology recommends the use of QEEG in ICU in the following situations [2, 40, 44]: patients at high risk of ischemic stroke, acute intracranial hemorrhage, vasospasm or severe intracranial hypertension; diagnosis and management of epileptic status in patients at high risk; titration of barbiturates; treatment with antiepileptic for non-convulsive causes; mannitol therapy for intracranial hypertension. Also, QEEG can be used to determine the appropriate time to turn off life support for a patient [45].

#### Learning and Attention Disorders

Many studies have emphasized the role of QEEG as a diagnosis tool in learning disorders [40, 46, 47], using spectral power and coherence, with an accuracy of 46-98% [48]. According to neurophysiology, the spectral power represents the sum of synchronous neuronal discharges [40]. The thickness of the cortical layer correlates positively with intelligence so that the EEG power may reflect the capacity of cortical information processing [40]. Recent studies using Low-Resolution Brain Electromagnetic Tomography Analysis (LORETA) reported a positive correlation between the intelligence quotient (IQ) and the increase in absolute power in bands α and β [49], a decrease of power in bands δ and θ [50] and a negative correlation between coherence and IQ especially in the frontal lobes [51, 52]. Generally, the higher the amplitude or absolute power is, the higher the IQ is [52, 53]. Instead, in the most severe learning disorders, the QEEG abnormalities are significant - the high value of the slow power is associated with a low IQ [54]. Other studies emphasized that coherence is positively correlated with IQ, being a real predictor of it [54, 55]. The American Association for Neuropsychiatry considers that QEEG may estimate the probability of a patient experiencing attention or learning disability based on repetitive studies [48].

Moreover, the American Association of Neurology recommends QEEG as an investigation for diagnosing learning disorders – Class II and III, Type D Recommendation [2, 44]. QEEG may play an essential role in the evaluation and treatment of attention deficit and hyperactivity disorder (ADHD), too. Children and adults diagnosed with ADHD show increased power in bands θ and δ; meanwhile, adolescents with ADHD have reduced β power compared to a control group [56–58]. The results of the meta-analysis published by Bresnahan and Barry suggest a pattern of ADHD on the Cz electrode (open eye, fixed sight): the θ/β ratio increased compared to the control group with a sensitivity of 86-90% and a specificity of 94-98% [59]. However, the results cannot be generalized, as changes in the θ/β ratio can be identified in other neuropsychiatric conditions. Along with audio-visual and cognitive tests, QEEG can be used to track therapeutic response and concentration performance in patients with ADHD [60].

#### Depression

QEEG plays a vital role in elucidating patterns of functional connections in patients with depression. Conventional EEG reveals abnormalities from 20 to 40% in patients with depression [40]. Even if the patterns are unspecific, QEEG could be a useful tool in the differential diagnosis between depression with minimal changes in EEG and severe functional or structural alteration [40]. The most common QEEG abnormalities in depression are presented in Table 2.

**Table 2:** QEEG abnormalities in depression.

α frontal asymmetry, a common marker associated with certain types of depression [61, 62]
Changes in frontal cordance [63, 64]
Asymmetry in the frontotemporal slow-wave [65]
Reduction of the interhemispheric coherence in the frequency bands δ and θ [66, 67]
Increasing of the absolute power in δ and θ bands in the right hemisphere [68]
Increase in θ in the posterior cerebral areas [69]
Changes in β activity [70–72]

The accuracy of these parameters in diagnosis has been verified in several studies, showing a sensitivity of 72% to 93% and a specificity between 75% and 88%, according to the American Association of Neuropsychiatry [48]. It recommends the use of QEEG as an additional method for the classification of unipolar and bipolar type and differential diagnosis between depression and healthy subjects, dementia, schizophrenia, and alcoholism [48]. Instead, the American Neurology Association classified QEEG as a Class II and Class III investigation, type D of recommendation [2, 44].

Frontal α asymmetry (FAA) is an essential marker of emotional responding and emotional disorders and could be measured as frontal asymmetry index or as a ratio between the difference and the sum of spectral power in F3 and F4 [73]. Although the relative differences are minor in FAA between patients with depression and healthy subjects, regardless of the method of calculating the FAA, some studies recommend the use of frontal asymmetry as a ratio and not as an index [74]. The argument is that if it does not divide by the sum (F4 + F3), there is a high probability of getting the frontal asymmetry as a negative value in both groups of patients [74].

A significant correlation has been suggested between the FAA and the behavioral activation system; the reduction in behavioral activation is associated with a predisposition for certain types of depression [74]. On the other hand, in major depression, the diagnostic role of the FAA is limited [74]. Thus, it has been shown that the FAA may have a prognostic value for diagnosing patients with psychopathological risk characterized by impairment of motivation mechanisms [75]. Moreover, the left FAA might associate with anhedonia, while the right FAA is identified in anxiety [74]. Further studies should focus on the role of the FAA in prognosis and monitoring of depression and less on the use of the FAA as a diagnostic tool.

Abnormalities of coherence and cordance were used to differentiate the unipolar depression from bipolar depression. Cordance is a mathematical combination of absolute and relative spectral power values along with each frequency band [76]. Also, cordance was correlated with regional cerebral blood perfusion and regional cerebral function in several studies [76,77]. Coherence in monitoring depression was generally measured by the method described by Thatcher in 1986 for TBI: in α and θ bands, the interhemispheric coherence (F3-F4, C3-C4, P3-P4, T7-T8), left interhemispheric coherence (F3-C3, F3-P3, F3-T5, C3-P3, C3-T5, P3-T5) and right interhemispheric coherence ( F4-C4, F4-P4, F4-T6, C4-P4, C4-T6, P4-T6) [76]. A synthesis of statistically significant results obtained from QEEG in patients with unipolar depression compared to patients with bipolar depressive disorder is presented in Table 3.

**Table 3:** QEEG markers in unipolar and bipolar depression.

Unipolar Depressive Disorder	Bipolar Depressive Disorder
Reduced interhemispheric coherence θ [76]	Reduced left α power [76]
	α frontal interhemispheric asymmetry [76]
	Increased β power [76]
	Increased left frontal α power [76, 78]
	α increased activation in the right temporal inferior and superior region, left occipital lobe and in the right precentral gyrus [79]
	Reduced α coherence in the right frontal and central regions and increasing α coherence in right parietal and temporal lobes [76]
	Increased θ coherence in the right central, parietal and temporal regions [76]

A meta-analysis based on articles published between 2000 and 2017 [80] assesses the accuracy of QEEG in predicting the response to antidepressant treatment and identifies the methodological limitations of QEEG analysis in depression. QEEG does not appear to be clinically relevant to monitoring the response to antidepressant therapy and is not yet recommended for the selection of psychiatric treatment [80].

#### Anxiety

FAA is associated not only with depression but also with anxiety. Patients with anxiety have a pattern of right frontal α activity higher than those without anxiety [81]. Patients with social phobia and those with panic attacks have a higher right frontal α activity [82, 83]. FAA correlates significantly with anxiety features [82, 83]. Parietotemporal asymmetry has also been reported in both anxiety and depressed subjects [81].

#### Dementia

QEEG abnormalities are usually identified in moderate and advanced stages of Alzheimer's disease. The most common changes are alterations of the δ and θ waves in the background activity and the reduction of the α-central frequency [84]. A reverse correlation between the stage of cognitive impairment and power in low-frequency bands was also reported [85]. Some studies emphasize a reduction in α and β activity [86, 87]. Furthermore, the α-like rhythm – a reduction in the α-frequency band in patients with mild Alzheimer's disease could be used as a diagnostic marker [88]. Coherence may quantify the hemispherical connectivity through the corpus callosum in the waking and sleeping state [89, 90] and reduced coherence both in patients with Alzheimer's disease and senile dementia was found [91].

Moreover, a decreased coherence in the θ, α, and β bands in the frontal and central areas was suggested compared to the control group [92]. According to the Brazilian Clinical Neurophysiology Society, frequency analysis may improve the diagnosis of slow waves, whereas combining QEEG with a cognitive scale is recommended to facilitate dementia diagnosis – type B of recommendation [40]. The role of QEEG in the diagnosis and assessment of dementia could be comparable to the utility of SPECT and MRI imaging techniques [40].

#### Other Neuropsychiatric Disorders

Spectral analysis is also useful in Parkinson's disease, providing an assessment of patients’ affective disorders [93]. Reduction of relative power δ, θ, α, β and absolute power θ, α, β in the anterior regions and interhemispheric asymmetry in θ, α, β bands with a right hemispheric activation were described in the literature [93]. A pilot study showed the potential of QEEG in diagnosing children with central auditory processing disorders. Changes of absolute power in δ, θ, low-β, and middle-β bands were suggested [94]. QEEG may also be used to differentiate subtypes of this pathology, but standards should be improved by future research [94]. QEEG is commonly used in the study of autism spectrum disorders, associating quantitative markers with changes in brain functions [95]. It can also be applied for therapeutic purposes using neurofeedback [95].

## Conclusions

QEEG represents a critical tool to improve clinical diagnosis and treatment response evaluation. Furthermore, several QEEG devices have been approved by the US Food and Drug Administration (FDA) for the post-hoc analysis of the digital EEG and are classified as Class II devices [40].

Although vast published literature on QEEG exists, this method is not known to be widely used, and there are still many scientific and controversial debates about its contribution to clinical practice. The causes of polemics in QEEG research are: the lack of methodology in managing the extensive database generated by EEG recordings – each specialist has its statistical analysis tools [40], inter- and intra-individual variability – the EEG is influenced by a number of biological factors (age, thickness of tissues, waking state), techniques (equipment, electrodes) and artifacts [40], the need of neurologists well-trained in QEEG interpretation and application to clinical practice [40]. Thus, the role of the QEEG is not necessarily to indicate a diagnosis immediately, but to be complementary to other investigations and to generate objective information in order to obtain a precise diagnosis, correct disease severity assessment, and specific treatment response evaluation.

## Conflict of Interest

The authors declare that there is no conflict of interest.
